# Detection of Buried Landmines in an Active Minefield Through Bioreporter-Based Optoelectronic Sensing of Their Chemical Signatures

**DOI:** 10.3390/bios16100539

**Published:** 2026-09-25

**Authors:** Yonatan Uziel, Mulkan Adgo, Natan Orlov, Roni Shohat, Shimshon Belkin, Aharon J. Agranat

**Affiliations:** 1 Department of Applied Physics, The Hebrew University of Jerusalem, Jerusalem 9190401, Israelagranat@savion.huji.ac.il (A.J.A.); 2 The Alexander Silberman Institute of Life Sciences, The Hebrew University of Jerusalem, Jerusalem 9190401, Israel; shimshon.belkin@mail.huji.ac.il

**Keywords:** bioreporters, optoelectronic sensors, biosensing

## Abstract

The research presented herein provides a first demonstration and a proof of concept of using sensing bacteria (bioreporters) to detect the chemical signatures generated by buried TNT-based landmines in an active minefield. The methodology, based on bioreporters generating a measurable signal in the presence of these chemicals, is fundamentally different from conventional approaches by detecting the explosive component of the device rather than its shape or metallic content. Furthermore, by virtue of the versatility of bioreporters to detect a broad range of target chemicals, the methodology can potentially be adapted to detect other types of explosives through the development of dedicated bioreporters. The measurements also highlight the potential of the biosensing modules to act as nodes in a sensor network for implementing the methodology across large areas. The methodology for detecting TNT via bacterial biosensing can be implemented in alternative configurations, enabling additional applications such as the identification of concealed TNT in cargo containers, buildings, and other environments.

## 1. Introduction

We demonstrate the detection of buried landmines, for the first time in an active minefield, using genetically engineered bacteria that sense chemical signatures emitted by the landmines into the surrounding soil. This study constitutes a proof of concept for bacterial sensing as a scalable and efficient approach for detecting buried landmines across large areas.

Buried landmines, remnants of past wars and military conflicts, pose a tremendous humanitarian and economic challenge worldwide [1], and the problem is worsening in regions where wars continue, most notably Ukraine [2]. The global count of these hidden hazards is estimated at 150 million, creating an acute and life-threatening risk for millions. In 2023 alone, landmines killed 5700 people, 84% of them civilians [3]. Typically, landmines are buried in minefields located at unmapped sites scattered across vast areas. Such “landmine-suspected” areas are estimated to cover hundreds of thousands of square kilometers worldwide [3]. However, within these suspected areas, the exact locations of the minefields are unknown. Therefore, the suspected areas in their entirety are treated as fully infested and are therefore unavailable for agriculture, mineral extraction, urban development, or infrastructure [4]. This situation underscores the urgent need for an efficient technology to detect and deactivate buried landmines. Cost-effective deactivation requires knowing where minefields are within the suspected areas [5], so that the deactivation efforts can be focused on actual hazards. Such technology is currently lacking. In addition, safely clearing suspected areas for civilian use requires a scalable method that can confirm the absence of buried mines across large tracts of land [6]. We present herein a conceptual basis for a comprehensive, cost-effective technology for detecting buried landmines based on bacterial chemical sensing, and we provide experimental evidence supporting its validity.

Bacterial sensing is a chemical-sensing methodology that uses engineered bacteria (bioreporters) as its core sensing elements [7,8]. These bioreporters are designed to produce a measurable signal when exposed to a specific target substance [9]. They are created by introducing into the bacteria a circular DNA molecule (plasmid) harboring a target-inducible gene promoter that acts as the sensing element, fused upstream to a reporter gene or a group of genes [10]. Upon exposure to the target substance, the promoter genes are expressed, driving the expression of the reporter genes, which in turn initiate the reporting mechanism [7].

In this study, the reporting mechanism is bioluminescence [11,12]; that is, the bioreporters emit light in response to their exposure to the target substance. Thus, the bioreporters function as autonomous, self-contained biochemical systems that detect the target and generate an optical signal to report its presence. It should be noted that even without being exposed to the target substance, the bioreporters spontaneously emit a weak background bioluminescence signal [13].

Employing bacterial sensing for detecting the locations of buried landmines is based on two underlying assumptions: (a) The buried landmines release specific chemicals that leak through their casing and permeate to the ground surface, forming there a “chemical signature” of the buried landmine [14,15]; and (b) This chemical signature can be sensed by the bioluminescent bioreporters that are dispersed in contact with the ground [16], and respond to sensing the chemical signature by emitting bioluminescent light [10,17,18].

In the research described herein, these assumptions were validated in a series of measurements using bioreporters that were dispersed on the terrain of an active minefield.

We argue that the results presented henceforth substantiate the validity of these assumptions, and as such provide the basis for employing bacterial sensing to detect buried landmines.

However, it should be recognized that exporting a bacterial sensing-based methodology of landmine detection from the research laboratory to operate in situ across wide areas outdoors is not straightforward. This relates in particular to the fact that the spatial distribution of the chemical signature generated on the terrain above buried landmines is influenced by a variety of parameters. These include the characteristics of the mine and its explosive content, burial depth and duration, soil type, relative humidity, and temperature. All of these factors affect the signal produced by the bioreporters in response to the chemical signature at the measurement location. Moreover, the heterogeneous nature of the terrain within a minefield causes the spatial distribution of the chemical signature generated on the terrain by the landmines to differ between landmines, irrespective of the fact that it is generated by identical landmines under similar environmental conditions. Consequently, this causes the signal to vary between measurement points, even when they are in close proximity to one another. Examples of these variations will be provided henceforth, and the scheme for establishing which of the measurements indeed indicates the presence of the chemical signature under the sensor footprint will be described and demonstrated.

We describe herein two sets of measurements of the chemical signature performed in an active minefield using the biosensing modules described earlier in [19]. We argue that these measurements substantiate the feasibility of employing bacterial sensing as the basis for a landmine detection methodology. We argue that, by virtue of the fact that the methodology is based on detecting the presence of the chemical signature generated by the chemical signature of the landmine, it is fundamentally different from conventional approaches by detecting the explosive component of the device rather than its shape or metallic content.

## 2. Materials and Methods

Using live bioreporter microorganisms as the core sensing elements in an outdoor bacterial sensing system requires providing conditions that support their survival. Moreover, as living biological entities, they require an appropriate casing that enables their integration into the optoelectronic system used to sample the bioluminescent signal they emit [17,20]. To address these requirements, the bioreporters were encapsulated in “sensing capsules”, small polymeric alginate spheres containing the bioreporters along with water and nutrients [21]. When deployed on the terrain, the bioreporters within the capsules are exposed to soil constituents, triggering light emission upon detection of the target material. The structural platform serving as a scaffold for the capsules is the capsule cassette, consisting of a narrow ring covered by a transparent circular window. A single layer of capsules is affixed beneath the window such that their lower portions protrude from the cassette. Thus, upon laying the cassette on the ground, the capsules are in contact with the soil, and the bioluminescent light they emit traverses through the cassette window. Further details regarding the properties of the sensing capsules and the capsule cassette are provided in the Supplementary Text S1.1.

The research focused on detecting buried landmines in which the explosive material was 2,4,6-trinitrotoluene (TNT), representative of ca. 90% of landmines worldwide. TNT is always accompanied by its primary breakdown product, 2,4-dinitrotoluene (DNT) [22,23]. Due to its volatility, DNT leaks through the landmine casing, migrates to the ground surface, and is generally considered to be a reliable chemical signature of buried TNT-based landmines. Hence, in potential, TNT landmines fulfill the first prerequisite condition for employing bacterial sensing to detect buried landmines, namely forming a chemical signature of the buried landmine on the surface of the ground. This is validated in the measurements described henceforth.

The measurements described herein employ a special strain of bioreporters that was genetically engineered to be sensitive to DNT. The TNT/DNT detection specificity of the employed bioreporters has been previously analyzed and verified [10]. We are therefore confident that the recorded BSM signals are indeed a direct and specific response to the traces of explosives diffusing out of the landmine casing.

The measurements were carried out using the biosensing modules (BSMs) previously described in [13], and schematically illustrated in Supplementary Figure S1a. Each BSM is an autonomous biosensing system that employs bacterial sensing to detect the presence of a target material within its footprint. The underside of each BSM is fitted with two capsule cassettes: an induced-signal cassette and a reference cassette. The capsules in the induced-signal cassette are in contact with the ground, enabling their bioreporters to respond to the target material. In contrast, the capsules in the reference cassette are isolated from the ground and produce spontaneous background bioluminescence. Further details of the BSM are provided in Supplementary Text S1.2. Each BSM contains two identical sensors (Figure S1b), each comprising an induced-signal channel and a reference channel, and outputs the difference in luminescence intensity between the two channels. A positive sensor output indicates the presence of the target material beneath the BSM footprint.

To increase confidence in target detection, the outputs of the two sensors within each BSM are plotted against each other. This constitutes the basis for the “Mirror imaging” criterion that is based on the assumption that the temporal dependence of the outputs of the two outputs of the adjacent sensors of the same BSM would be similar. Positive detection is confirmed when the outputs of the two sensors exhibit mirror symmetry. The effectiveness of the “mirror imaging” criterion was demonstrated in measurements conducted in a sandbox containing 5 g of DNT buried at a depth of 5 cm for 5 years. A BSM loaded with bioreporters from the same batch in both cassettes was placed on the sand surface, and the outputs of the two sensors were monitored in parallel (Figure 1). Further details regarding the Mirror imaging criterion are provided in the Supplementary Material Text S1.3.

## 3. Results

The feasibility of detecting buried landmines using bacterial sensing was investigated through measurements conducted using a set of BSMs deployed in a minefield containing active antipersonnel TNT landmines.

Prior to the field measurements, the DNT detection sensitivity of the bioreporter strain used in the experiments was evaluated in a laboratory experiment. As shown in Supplementary Text S1.4, a BSM loaded with this bioreporter strain detected a DNT concentration of 30 ppb in an aqueous solution. Further details are provided in Supplementary Text S1.4 and Supplementary Figure S2.

The active minefield experiments were carried out in the Ein-Sukkot minefield, located in the Jordan Valley, Israel. The minefield consists of rows of antipersonnel landmines that were buried approximately 57 years ago. Photographs of the type of antipersonnel landmines buried in Ein-Sukkot are presented in Figure 2a,b.

These landmines consist of rectangular Bakelite boxes (13.5 × 6.7 × 5.6 cm) containing a 200 g cast TNT “brick” at one end and an activation mechanism at the opposite end. The landmines are triggered by applying vertical pressure to the ground above the activation mechanism [24]. The measurements were conducted in a segment of the Ein Sukkot minefield approximately 12 m wide and 30 m long. The inspected segment was located adjacent to a mine-free area, which served as a control site.

A special mobile platform was developed to enable the safe deployment of the BSM units. Figure 2c presents a side view of the inspected segment together with the deployment platform. The platform consisted of a metallic bridge mounted on four wheels and supporting a flat structure suspended approximately 1 m above the ground and extending across the width of the inspected area. The two western wheels were positioned outside the fence, while the two eastern wheels rested within the cleared area, allowing the platform to move safely along the inspected segment. The inspected area and the deployment platform are presented in Figure 2c.

Prior to conducting measurements inside the minefield, a negative-control measurement was performed by deploying a BSM in the cleared area outside the minefield. As shown in Figure 3, the signal level generated in this measurement is significantly lower than the signal level measured in proximity to the landmines. Furthermore, the signal reaches its maximum level within less than 1 h, which is characteristic of measurements generated by BSMs that are positioned on clear soil so that their bioreporters produce only spontaneous emission. This confirmed the absence of detectable DNT on the terrain inspected by the BSM in the cleared area.

Before deploying the BSMs in the minefield, the inspected area was scanned with a metal detector to identify the locations of the metallic activation mechanisms of the buried landmines. It should be noted that the metal detector identifies the location of the activation mechanism, whereas the TNT charge that generates DNT is located at the opposite end of the landmine casing, 13 cm from the mechanism. Furthermore, the orientation of the landmine casing relative to the ground is unknown. In principle, the orientation of the landmine could be determined by manually exposing it after the measurements; however, this is prohibited by safety regulations.

The measurements were aimed at determining whether the chemical signature of a buried landmine could be detected by BSMs on the ground surrounding the landmine. To this end, the BSMs were deployed directly above selected landmines and at various distances around them. In the second set, the BSMs were deployed randomly within a segment of the minefield where landmines had been detected, but their exact locations were unknown.

More specifically, prior to deploying the BSMs, two of the landmines detected by the metal detector were selected, and two sets of four BSMs, each attached to an equilateral triangular scaffold (Figure 4a), were positioned above them. The scaffolds were positioned so that one BSM was located directly above the landmine activation mechanism at the center of the triangle, while the other three BSMs were positioned at the triangle vertices. In one scaffold, the distance between the center and the vertices was 30 cm, whereas in the second scaffold, it was 45 cm. Each BSM was enclosed by a darkening skirt that prevented ambient light from penetrating its interior. The skirts also shielded the sensing capsules from direct sunlight, thereby preventing them from heating. The BSMs were numbered as indicated in Figure 4b.

The output signals produced by the four BSMs, with a distance of 30 cm between the central BSM and each of the BSMs positioned at the triangle vertices, are presented in Figure 5. While the BSMs positioned at the triangle vertices clearly indicated DNT detection within their respective footprints, the central BSM showed no response. This may be attributed to its position directly above the activation mechanism, which was located 13 cm from the TNT charge. Because the landmines in the tested minefield were buried close to the surface, TNT vapors released from the landmine casing had to permeate laterally through the surrounding soil to reach the bioreporter capsules in the BSMs. In addition, the orientation of the landmine may have resulted in the TNT charge being positioned closer to BSM3 and BSM4, which could explain the lower signal generated by BSM2.

The measurements of the four BSMs attached to the triangular scaffold in which the distance between the central BSM and the BSMs at the vertices was 45 cm yielded similar results. Namely, the BSMs at the vertices detected DNT within their footprint, whereas the central BSM did not. It should be noted that, in both cases, the temporal dependence and intensity of the bioluminescence varied among the BSM locations, likely reflecting the heterogeneous nature of the soil surrounding the buried landmine. Nevertheless, the mirror-imaging criterion was found to be an effective qualitative criterion in confirming the positive detection of the chemical signature. These results were augmented with additional measurements in which two adjacent rows of BSMs were deployed orthogonally to the rows of the buried landmines. The results of these measurements indicated a posteriori that two landmines were buried in the area covered by the deployed BSMs. The results obtained in the BSMs in both triangles and the two rows are summarized in Table 1.

## 4. Discussion

The research presented herein examined the potential of bacterial sensing as the basis for a generic methodology for detecting buried landmines. As stated above, the methodology derived from this concept relies on two prerequisite conditions: (a) the explosive material in buried landmines generates a chemical signature that permeates the surrounding terrain; and (b) the sensitivity of the bioluminescent bioreporters is sufficient to respond to this signature by emitting a measurable optical signal. These conditions were investigated in an active minefield containing antipersonnel TNT landmines. To this end, the experiments employed biosensing modules (BSMs) equipped with cassettes of bioreporter capsules affixed to their undersides. The BSMs were placed on the ground, enabling them to detect the presence of the chemical signature beneath their footprints through the sensing of DNT by the bioreporters within the capsules. We argue that the results of these measurements substantiate the validity of both prerequisite conditions. Moreover, these in situ experiments provide a proof of concept for scaling bacterial sensing from the detection of the chemical signature at individual locations to the large-scale localization of buried landmines across a minefield. Such scaling could be achieved by deploying a network of BSMs over wide areas, thereby enabling distributed sensing of the terrain for the presence of landmine-derived chemical signatures.

The in situ experiments described in this communication are subject to two limitations, arising from the necessity of conducting the study in a live minefield and the limited number of available BSMs. These are, on the one hand, the limited number of experimental replicates and, on the other, the lack of independent chemical-analytical corroboration of the presence of DNT at the assayed locations. However, as pointed out in the introduction, the TNT/DNT detection specificity of the employed bioreporters has been previously characterized and experimentally verified. We are therefore confident that the BSM signals recorded in the present study represent a direct and specific response to traces of explosive-related compounds diffusing from the landmine casing into the surrounding terrain. It should also be emphasized that the objective of this study was not to provide a statistically comprehensive field validation, but rather to establish a proof of concept for an innovative approach to buried-landmine detection. In this respect, the results demonstrate the feasibility of detecting the chemical signatures of buried landmines in situ using bacteria-based sensing modules and provide a clear basis for advancing the methodology toward larger-scale field validation and, ultimately, a mature landmine-detection technology.

Landmine detection based on bacterial sensing differs fundamentally from conventional detection methods, including metal detectors [25], ground-penetrating radar [26], acoustic waves [27], and thermal cameras [28]. These methods rely on detecting physical characteristics associated with the landmine’s casing, metallic components, or shape, thereby providing only indirect indications of the presence of a buried landmine [29]. Consequently, they are susceptible to both false-positive and false-negative detections [30]. For example, metal detectors cannot distinguish between landmines and other metallic debris in the surrounding area, potentially resulting in false positives. Conversely, landmines containing little or no metal may be effectively undetectable by such systems, resulting in false negatives. In contrast, bacterial sensing provides a fundamentally different approach by directly detecting the chemical signature of the explosive and thus offering a direct indication of the presence of a buried landmine [10].

From a systems-engineering perspective, such a methodology implicitly assumes that the locations of buried landmines are correlated with the spatial distribution of their chemical signatures across the inspected area, and that this distribution can be mapped by bacterial sensing. Put differently, the methodology employs bacterial sensing to map the spatial distribution of the chemical signature across the inspected area and subsequently infer the locations of buried landmines from the resulting map.

This process comprises three stages: sensing, sampling, and data processing. Sensing is performed by the bioreporters, which function as optoelectronic sensing elements that detect the presence of the chemical signature in their immediate vicinity. Sampling is performed by the sensor’s optoelectronic circuitry, which acquires the signals generated by the bioreporters, converts them into digital signals, and transmits them to a central processing unit. Data processing then involves aggregating the signals acquired by the optoelectronic sensors across the inspected area and using the resulting dataset to generate a map of the spatial distribution of the chemical signature associated with the buried landmines. The locations of the buried landmines can subsequently be inferred from this spatial map.

The experiments also demonstrate that the BSMs used in these measurements are well suited to serve as nodes in an outdoor sensor network performing the sensing and sampling stages of the proposed methodology. In particular, the BSMs incorporate several features that support such deployment: (i) a design intended for in situ operation, with an operational period of up to 12 h; (ii) the capability for repeated operation; (iii) day-and-night operation in a “closed-architecture” configuration, independent of ambient illumination; (iv) operation over a temperature range of approximately 21–35 °C, consistent with the temperature requirements of the employed bioreporters. An advanced BSM currently under development incorporates a heated window adjacent to the capsule cassette, which is expected to extend the operational temperature range to approximately 12–35 °C; and (v) the use of the “mirror-imaging” decision criterion, which facilitates reliable detection over a wide signal dynamic range and thereby supports operation in minefields exhibiting substantial spatial variation in signal intensity.

As mentioned earlier, the intensity of a landmine’s chemical signature and its spatial distribution may be influenced by a variety of parameters, including the type of mine and its explosive content, burial depth and duration, soil type, relative humidity, and temperature. Consequently, the operational conditions of the BSM network must be adapted to the specific characteristics of the minefield under investigation, as well as to the environmental and climatic conditions prevailing in the deployment area [31]. This adaptation can be achieved by tuning two key parameters of the network: the detection sensitivity of the BSMs and the spatial resolution of the deployed network. The sensitivity of the BSMs can be adjusted by varying the number of capsules in their cassettes, which can be achieved by selecting an appropriate cassette size for each BSM. The spatial resolution of the network, in turn, is determined by the number of BSM units deployed per unit area. The sensitivity and spatial resolution appropriate for a given area of investigation can be determined by first measuring the chemical signature in a small representative segment of the inspected area, using BSMs configured for high sensitivity and deployed at high spatial density. The optimal sensitivity and spatial resolution for the entire area can then be derived from these measurements and implemented in the deployed network.

## 5. Conclusions

In conclusion, the research presented herein provides a proof of concept for the underlying principle of a methodology for detecting TNT landmines by demonstrating that bioreporters generate measurable signals in response to the chemical signatures produced by landmines in their vicinity. This methodology is unique in that it detects the chemical signature produced by the TNT contained within the landmine, rather than relying on the landmine’s shape or the metallic content of its casing.

Furthermore, the measurements highlight the potential of BSMs to function as nodes in a sensor network implementing the methodology for mapping chemical signatures associated with landmines buried across wide areas. The methodology may also be implemented in different configurations, enabling additional applications, such as the detection of concealed TNT in cargo containers, buildings, and other environments. This capability is particularly relevant to the detection of unexploded ordnance (UXO) containing TNT.

Finally, because bioreporters can be genetically engineered to respond to different chemical targets, the methodology is inherently generic and can be adapted to map traces of different target substances, including other explosives [32] and additional compounds of interest. Moreover, it provides a basis for applications in other domains, such as precision agriculture and environmental monitoring.

## Figures and Tables

**Figure 1:**
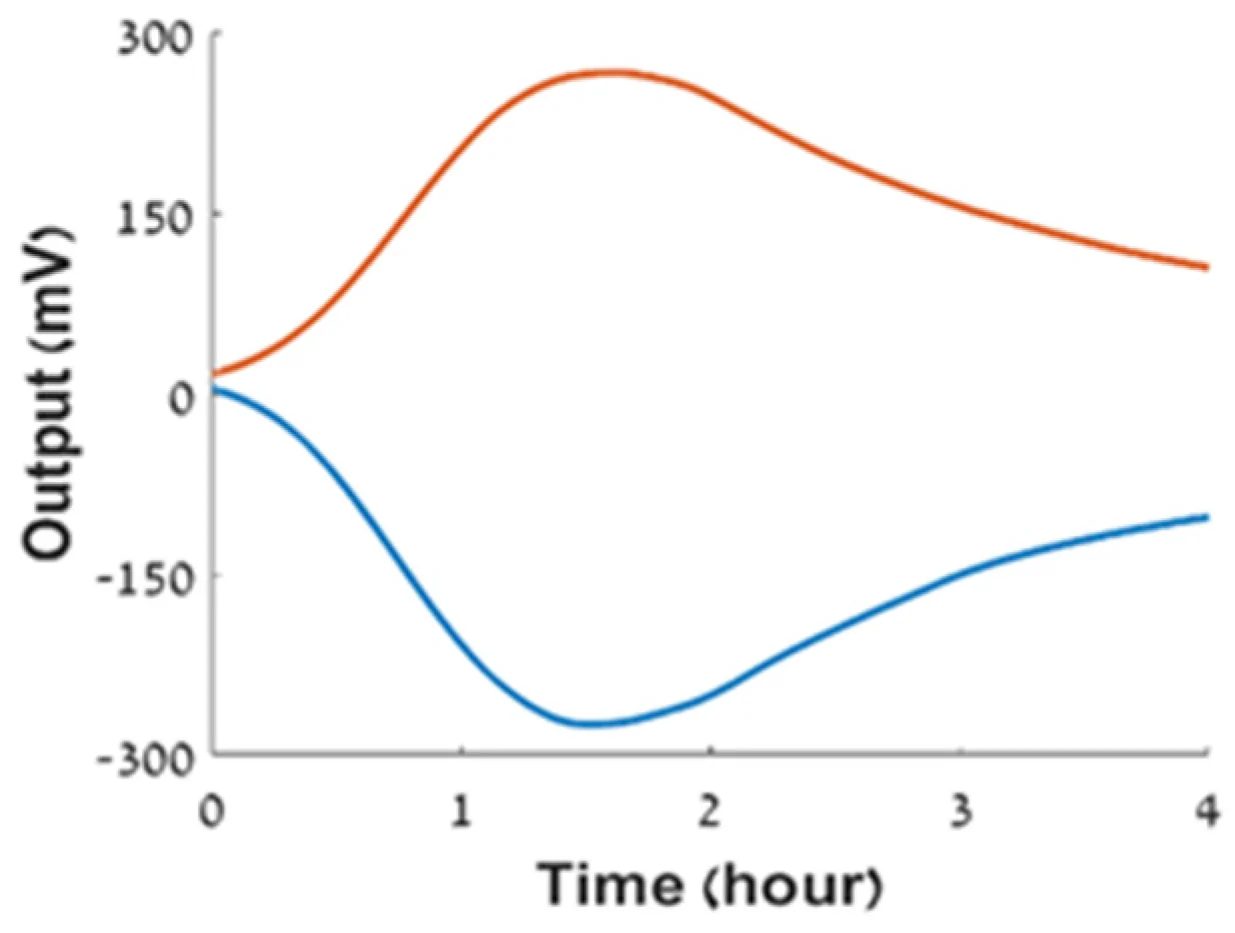
Demonstration of the “Mirror imaging” criterion, showing the outputs of the two sensors of a BSM positioned on top of a lump of DNT weighing 5 g buried in sand 5 cm below the surface. The outputs are plotted against each other, exhibiting mirror symmetry.

**Figure 2:**
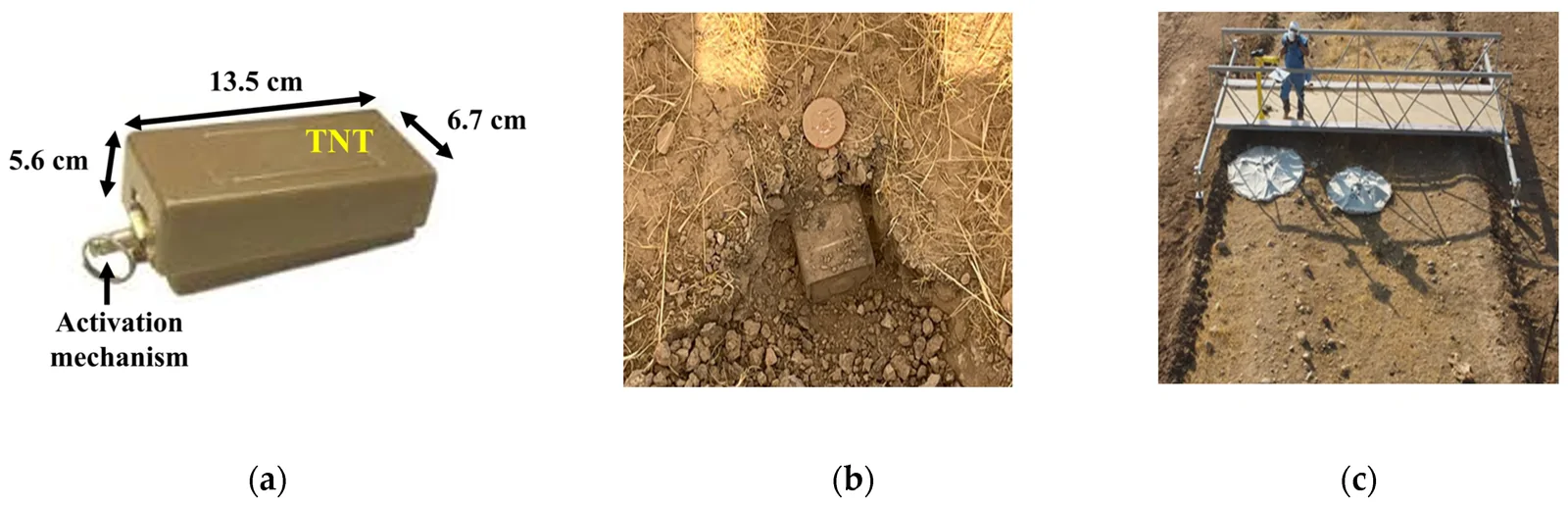
(**a**) A photograph of an Mk. 4 antipersonnel landmine; (**b**) An image of an Mk. 4 antipersonnel landmine buried in the Ein Sukkot minefield and partly exposed; (**c**) Image of the inspected area and the deployment platform.

**Figure 3:**
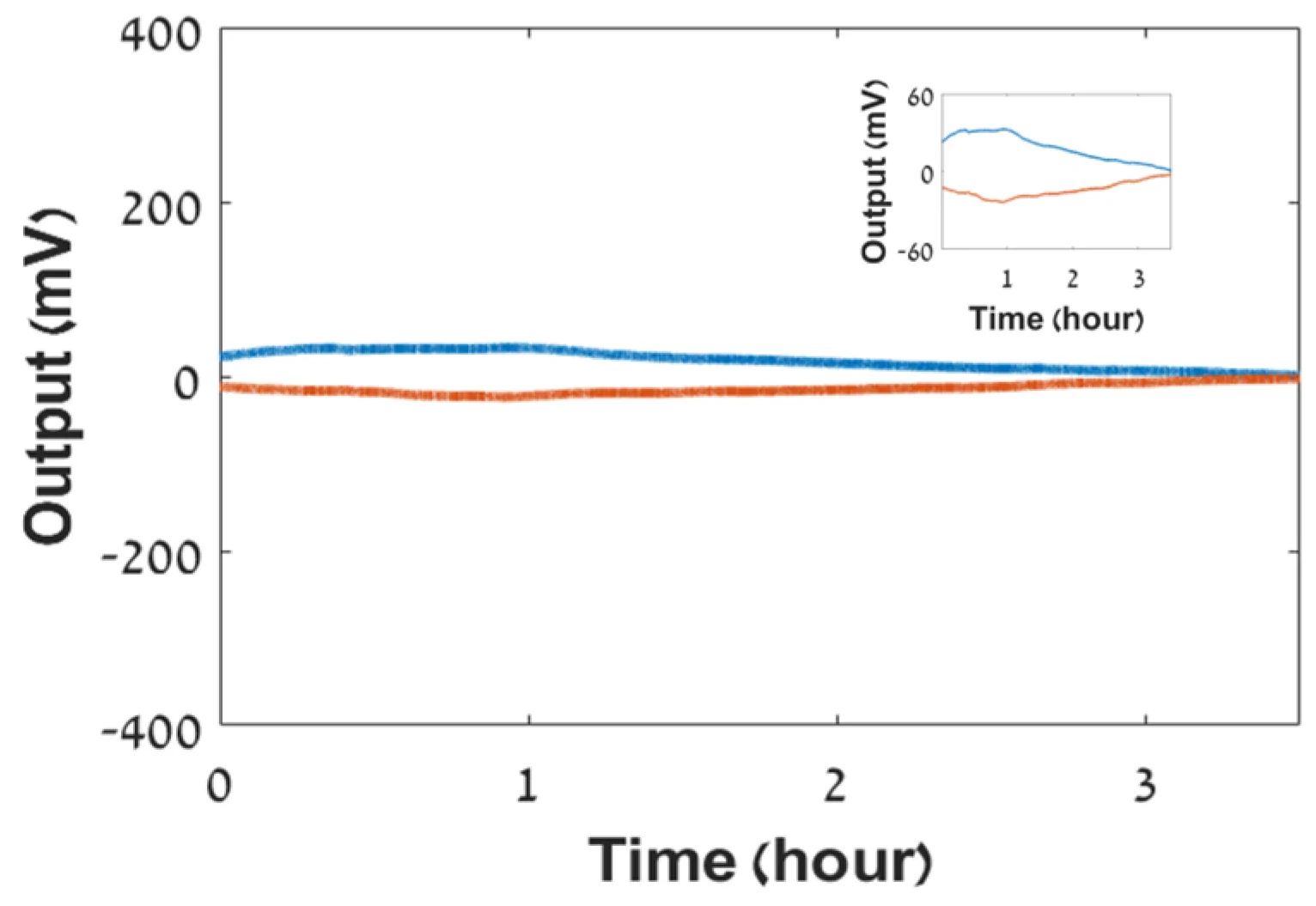
A negative control measurement using a BSM positioned in the cleared area outside the minefield. The BSM was loaded with the same bioreporter strain and carried out under identical environmental conditions as in the measurements inside the minefield.

**Figure 4:**
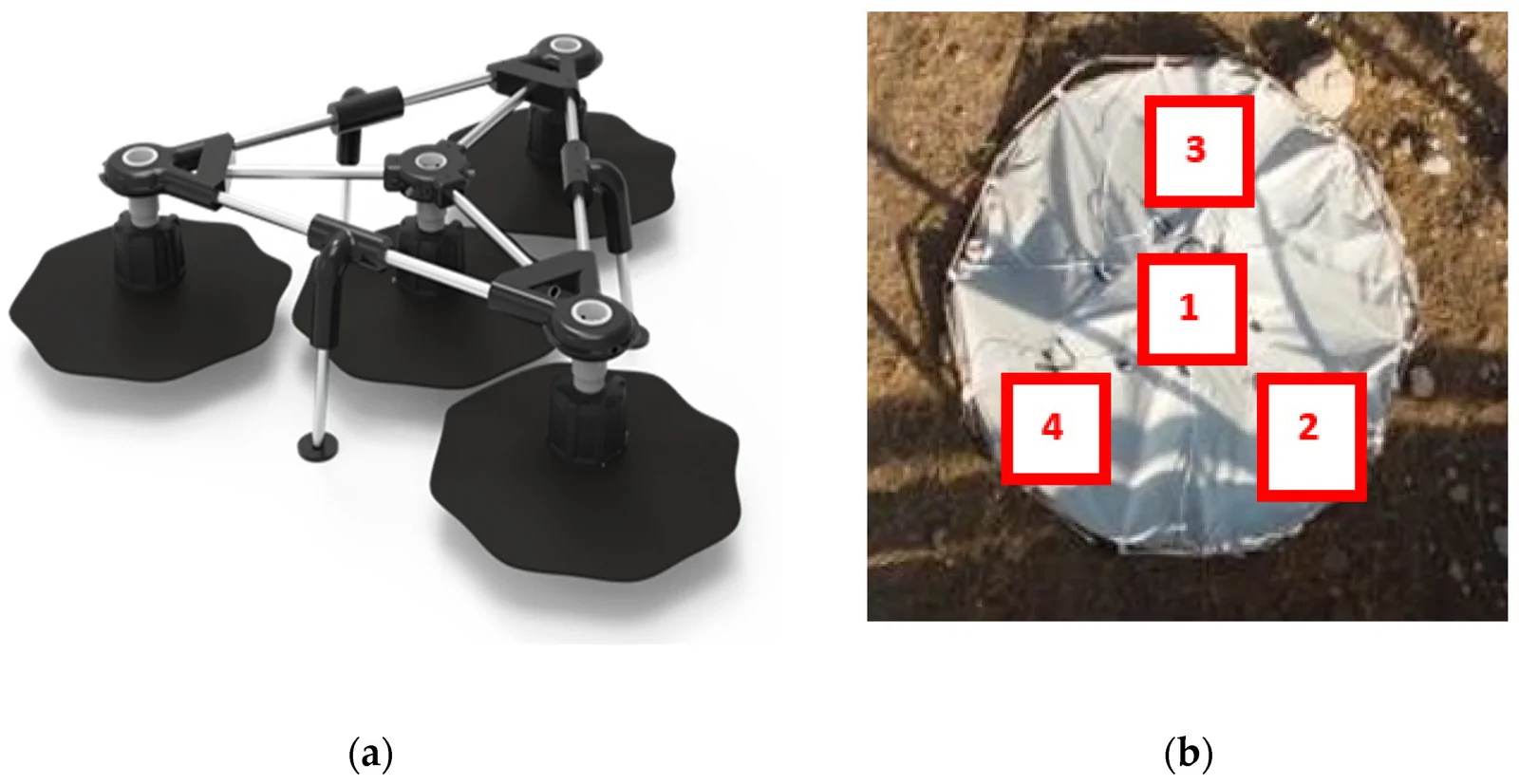
The four BSMs attached to the triangular structure: (**a**) side view showing one BSM at the center and 3 BSMs at the vertices that were 30 cm from the center; (**b**) Top view showing the platform covered with the white skirt, and the numbers assigned to the BSMs.

**Figure 5:**
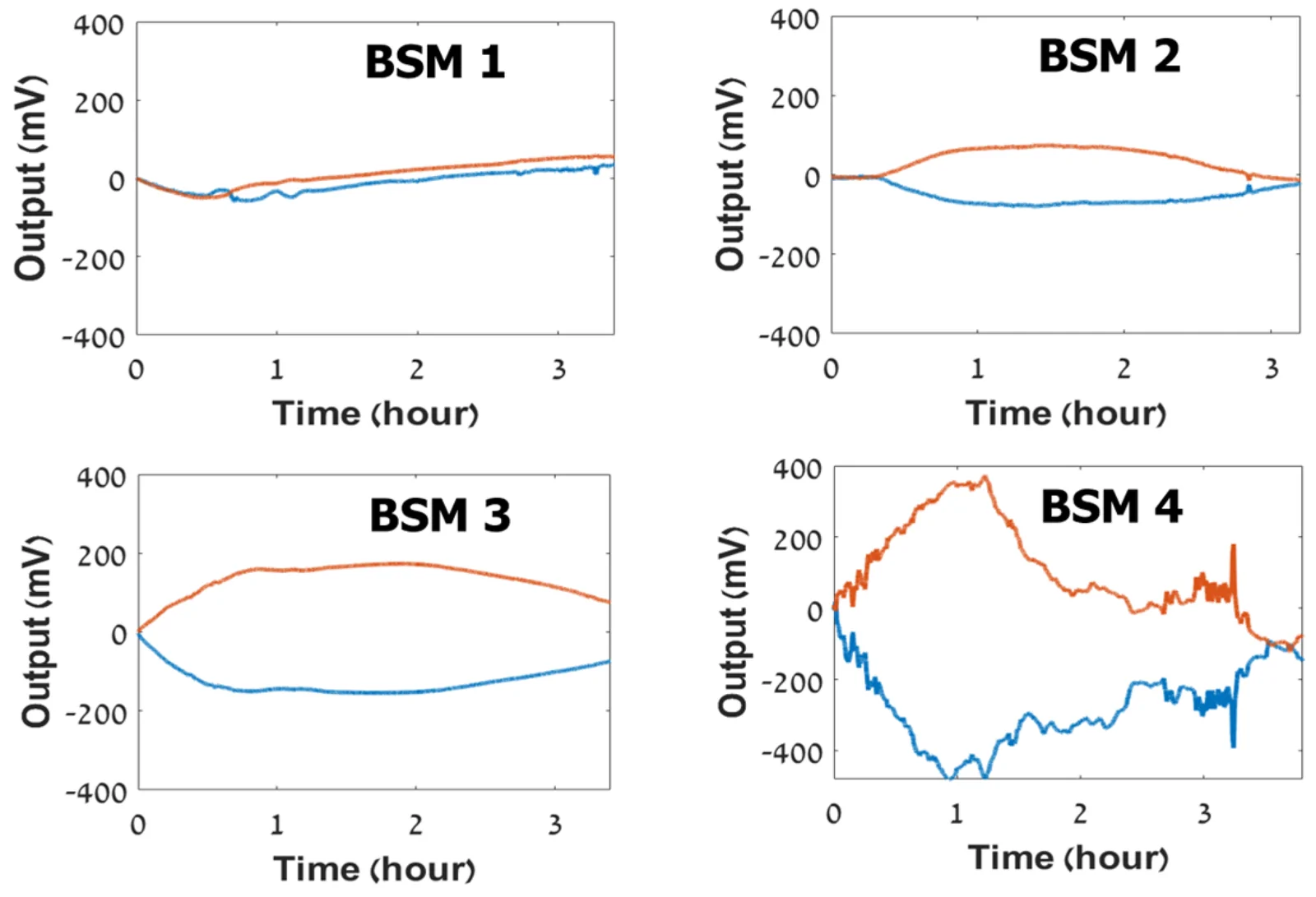
Time dependence of the outputs of the 4 BSMs was carried out using the deployment triangle shown in Figure 4a.

**Table 1:** Summary of detection results in the Ein Sukkot minefield.

Peak Value [mV]	BSM Number	Detection	Distance to the Landmine [cm] (**)	Measurement Number (*)
31	BSM 1	No	0	1
98	BSM 2	Yes	30	2
162	BSM 3	Yes	30	3
380	BSM 4	Yes	30	4
16	BSM 1	No	0	5
230	BSM 2	Yes	45	6
151	BSM 3	Yes	45	7
47	BSM 4	Yes	45	8
110	BSM 1	Yes	15	9
100	BSM 2	Yes	15	10
180	BSM 3	Yes	15	11
10	BSM 4	No	15	12
5	BSM 6	No	15	13
53	BSM 5	Yes	45	14

(*) 30 cm triangle; measurements 1–4. 45 cm triangle; measurements 5–9. Two rows; measurements 10–14. (**) For both scaffolds, BSM No.1 was positioned above the activation mechanism.

## Data Availability

Data will be provided upon reasonable email request by the corresponding author.

## Supplementary Materials

The following supporting information can be downloaded at: https://www.mdpi.com/article/10.3390/bios16100539/s1: Text S1.1: The sensing capsules and the capsule cassette; Text S1.2: The biosensing module; Text S1.3: The “mirror image” criterion; Text S1.4: The sensitivity of the bioreporters; Figure S1: The biosensing module; Figure S2: The temporal dependence of the bioluminescence signal. References [33,34,35,36] are cited in Supplementary Materials.
