# Supplementary material for: Detection of Buried Landmines in an Active Minefield Through Bioreporter-Based Optoelectronic Sensing of Their Chemical Signatures
Source: Biosensors (Basel). 2026 Sep 25;16(10):539. doi: 10.3390/bios16100539 (PMC13702335; doi:10.3390/bios16100539)
Supplement: Supplementary file 1 [file biosensors-16-00539-s001.zip › biosensors-4522552-supplementary.pdf]

biosensors

MDPI

Article

Detection of Buried Landmines in an Active Minefield Through Bioreporter-Based Optoelectronic Sensing of Their Chemical Signatures

Yonatan Uziel [1,*], Mulkan Adgo [1], Natan Orlov [1], Roni Shohat [1], Shimshon Belkin [2] and Aharon J. Agranat [1]

1 Department of Applied Physics and the, The Hebrew University of Jerusalem, Jerusalem 9190401, Israel; mulkan.adgo@mail.huji.ac.il (M.A.); natan.orlov@mail.huji.ac.il (N.O.); roni.shohat@mail.huji.ac.il (R.S.); agranat@savion.huji.ac.il (A.J.A.)
2 The Alexander Silberman Institute of Life Sciences, The Hebrew University of Jerusalem, Jerusalem 9190401, Israel; shimshon.belkin@mail.huji.ac.il
* Correspondence: yonatan.uziel@mail.huji.ac.il

S1. Supplementary Text

S1.1. The Sensing Capsules and the Capsule Cassette

Live bioreporters require a hydrated environment that provides water and nutrients for viability. To satisfy this requirement, the bioreporters were encapsulated in “Sensing capsules” containing both water and nutrients. These capsules sustain bioreporter viability for up to ~12 h following deployment on the ground, during which the cells emit bioluminescence in response to sensing the chemical signatures released from buried landmines and diffuse into the surrounding soil [1].

The sensing capsules are alginate-based polymeric spheres with a diameter of approximately 4 mm. Each capsule typically contains 0.5–1 × 10^6 bioreporter cells, together with water and nutrient medium (Lysogeny Broth[2], (LB)). The capsules can be mass-produced using standard bioprocessing methods and stored and transported at 4°C for 6–8 weeks with negligible loss of sensing performance [3].

Prior to use, the refrigerated capsules undergo a refresh step consisting of a 2 h stirring at 37°C in LB [4]. The capsules are then loaded into open cassettes that serve as operational platforms. These “Capsule cassettes” are affixed to the underside of the sensing system such that, upon placement on the ground, the capsules establish direct contact with the soil within the unit footprint.

In this configuration, the encapsulated bioreporters generate a bioluminescent signal upon exposure to the target analyte beneath the sensing unit footprint. Accordingly, the capsules both maintain bioreporter viability under field conditions and serve as optoelectronic sensing elements integrated into the frontend circuitry of the sensing unit.

S1.2. The Biosensing Module

The sensing system that was used to detect the presence of the chemical signature produced by the buried landmines on the ground is the “biosensing module” (BSM), described in detail in [5].

The BSM is an autonomous optoelectronic sensing system designed to be positioned on the ground outdoors and report the presence of the target material in the soil under its footprint. A schematic illustration of the BSM is presented in Supplementary Fig. S2a. Two half-circle cassettes, each containing a single layer of capsules, are affixed to the underside of the BSM. In the induced-signal cassette, the capsules touch the soil underneath the BSM,

causing the bioreporters in them to generate a bioluminescent signal induced upon sensing the target material in the soil. In the reference cassette, the capsules are detached from the ground, so that the bioreporters in them generate only a weak spontaneous bioluminescent signal. As shown in Supplementary Fig. S2a, for obtaining improved statistics, each BSM is equipped with two identical sensors that operate independently. A schematic illustration of the sensors is presented in Supplementary Fig. S2b. Each sensor consists of an induced-signal channel and a reference channel. The induced-signal channel samples half of the capsules in the induced-signal cassette, whereas the reference channel samples half the capsules in the reference cassette. The outputs of the induced-signal and reference channels are compared by a differential amplifier, which produces at the output of the sensor an analog signal that represents the difference between them. An indication of the presence of DNT under the footprint of each sensor is when the induced-channel signal is stronger than the reference signal. The analog outputs of the two differential amplifiers are digitized by the digital unit and transmitted by Wi Fi to the central control unit of the BSM network.

The bioreporters in the capsules commence functioning upon completing the refresh period. A typical example of the temporal dependence of the signal at the output of the induced-signal channel is presented in Supplementary Fig. S1. As shown, upon sensing the presence of the target material in their vicinity, the signal at the output of the induced-signal channel gradually increases, reaches a maximum, and then decays to zero. The temporal dependence of the signal depends on the bioreporter batch and the environmental conditions that prevail during the measurement (typically reaching the maximum level after 1.5 to 2 hours).

As indicated, the BSMs are designed to operate in different minefields. It was observed that even in the same minefield, signals produced in different measurements vary in strength and temporal behavior due to differences between the bioreporter batches in the capsules. Additionally, the response of the bioreporters is also affected by the variations in the environmental conditions prevailing during the measurements, and the location of the BSMs across the inspected minefield. This necessitated defining a decision criterion for determining whether a measurement indicates the presence of DNT under the BSM footprint.

S1.3. The "Mirror Image" Criterion

The criterion that was adopted to establish confidence in detection of the chemical signature by the BSM is the “mirror imaging” criterion. It is based on the assumption that in each measurement, the output signals of the two adjacent sensors in the same BSM are expected to exhibit similar temporal dependence. This assumption was established in many measurements with different bioreporter batches that were carried out under different environmental conditions. These measurements substantiated the underlying assumption, namely, that the signal produced by the two sensors in the same BSM has similar temporal dependence.

To evaluate the similarity between the two outputs, the output of one sensor was inverted, and the two outputs were then plotted against each other. For a positive detection of the chemical signature (DNT) beneath the BSM footprint, the two sensors are expected to produce output signals that are mirror images of each other.

The effectiveness of the “mirror imaging” criterion in field measurements was tested outdoors by comparing measurements of the chemical signatures produced by antipersonnel landmines buried at the same location in the desert, but conducted at different times of the year. The first measurement was conducted in July, following several weeks of very hot weather, with temperatures ranging from 26°C to 41°C in the shade and continuous exposure of the ground to direct sunlight throughout the day. The second

measurement was conducted at the same location approximately eight weeks later, in September, when temperatures ranged from 18°C to 31°C.

In the July measurement, the signals of the two sensors in the BSM reached maximum values of approximately 1 mV and −1 mV, respectively. In contrast, in the September measurement, the corresponding signals reached maximum values of approximately 55 mV and −40 mV, respectively. And yet, although the signal levels differed by a factor of approximately 50 between the two measurements, the temporal dependence of the signals generated by the two sensors in the BSM was similar in both cases. According to the mirror imaging criterion, this temporal correspondence indicated reliable detection of DNT beneath the BSM footprint.

A possible explanation for the substantial difference in signal levels between the two measurements is attributed to the intense solar radiation incident on the terrain prior to the July measurement, which presumably caused rapid evaporation and depletion of DNT from the ground surface. Consequently, the July measurement produced a near-zero signal response. Nevertheless, the two sensors exhibited similar temporal responses, consistent with the mirror imaging criterion. The substantially stronger signals obtained in September presumably reflect the cooler environmental conditions which prevailed in the days before the measurement. This allowed the chemical signature produced by the landmine to reaccumulate at the ground surface.

These observations also indicate that the DNT detected in September had accumulated at the ground surface after the July measurement, rather than representing DNT that had remained there continuously since the landmines were buried. The near-zero response observed in July suggests that the surface DNT concentration had been reduced to nearly zero by the preceding period of intense solar irradiation. The subsequent accumulation of DNT at the surface therefore occurred during the approximately eight-week interval between the two measurements.

It should be borne in mind that, at the present stage of the research, mirror imaging is used as a qualitative criterion for substantiating the underlying principle of the proposed methodology. Converting the mirror imaging criterion into a quantitative detection criterion will be addressed in the next phase of the project, when a substantially larger number of BSMs will be available. The expanded BSM network that will be deployed will enable the generation of a sufficiently large database of measurements, which is a prerequisite for developing and validating quantitative criteria for DNT detection.

S1.4. The Sensitivity of the Bioreporters

Prior to conducting the field measurements, the sensitivity of measuring small concentrations of DNT on the ground was tested in a laboratory experiment. The measurements were conducted in a climate cell that was stabilized to operate at 25ºC and a humidity of 50%. A tray with 25 samples was placed in the cell. Each sample was a Petri dish containing sand mixed with varying concentrations of DNT. The samples were covered with a single layer of capsules containing bioreporters. The genetic engineering details of these bioreporters are given in [6]. The bioluminescence generated by the bioreporters in response to sensing DNT in the respective samples was captured by a scientific CMOS camera (pco.edge 5.5 CLHS). A set of typical results is depicted in Supplementary Fig. S1. As shown, the bioluminescence signal generated by the sample containing 30 ppb of DNT in the sand (i.e., 30 μg of DNT per 1 kg of sand) was significantly stronger than the bioluminescence signal produced spontaneously by the sample that contained only sand.

S2. Supplementary Figures

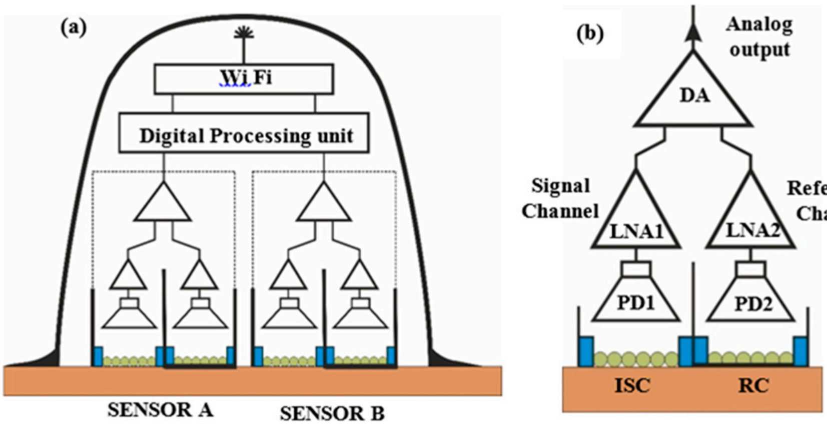

Figure S1. The biosensing module. (**a**) Schematic illustration of the BSM with its two sensors; (**b**) Schematic illustration of the analog circuit of one sensor in the BSM with its induced-signal and reference channels and their sensing cassettes: (PD) Photodiode, (LNA) Low-noise amplifier, (DA) Differential amplifier.

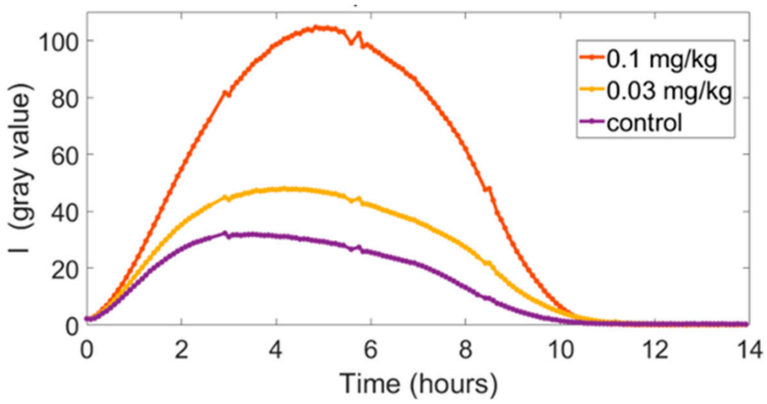

Figure S2. The temporal dependence of the bioluminescence signal generated by capsules deposited on samples containing sand mixed with traces of DNT at different concentrations.

References

1. Riseh, R.S.; Skorik, Y.A.; Thakur, V.K.; Pour, M.M.; Tamanadar, E.; Noghabi, S.S. Encapsulation of Plant Biocontrol Bacteria with Alginate as a Main Polymer Material. *Int. J. Mol. Sci.* **2021**, *22*, 11165, https://doi.org/10.3390/ijms222011165.
2. Sezonov, G.; Joseleau-Petit, D.; D'ARi, R. *Escherichia coli* Physiology in Luria-Bertani Broth. *J. Bacteriol.* **2007**, *189*, 8746–8749, https://doi.org/10.1128/jb.01368-07.
3. Bassani, J.C.; Santos, V.A.Q.; Barbosa-Dekker, A.M.; Dekker, R.F.; da Cunha, M.A.A.; Pereira, E.A. Microbial cell encapsulation as a strategy for the maintenance of stock cultures. *LWT* **2019**, *102*, 411–417, https://doi.org/10.1016/j.lwt.2018.12.058.
4. Shahzadi, I.; Al-Ghamdi, M.A.; Nadeem, M.S.; Sajjad, M.; Ali, A.; Khan, J.A.; Kazmi, I. Scale-up fermentation of Escherichia coli for the production of recombinant endoglucanase from Clostridium thermocellum. *Sci. Rep.* **2021**, *11*, 1–10, https://doi.org/10.1038/s41598-021-86000-z.
5. Agranat, A.J.; Kabessa, Y.; Shemer, B.; Shpigel, E.; Schwartsglass, O.; Atamneh, L.; Uziel, Y.; Ejzenberg, M.; Mizrachi, Y.; Garcia, Y.; et al. An autonomous bioluminescent bacterial biosensor module for outdoor sensor networks, and its application for the detection of buried explosives. *Biosens. Bioelectron.* **2021**, *185*, 113253, https://doi.org/10.1016/j.bios.2021.113253.
6. David, L.; Shpigel, E.; Levin, I.; Moshe, S.; Zimmerman, L.; Dadon-Simanowitz, S.; Shemer, B.; Levkovich, S.A.; Larush, L.; Magdassi, S.; et al. Performance upgrade of a microbial explosives' sensor strain by screening a high throughput saturation library of a transcriptional regulator. *Comput. Struct. Biotechnol. J.* **2023**, *21*, 4252–4260, https://doi.org/10.1016/j.csbj.2023.08.017.
